# Comment on “Rheumatoid Arthritis in Agricultural Health Study Spouses: Associations with Pesticides and Other Farm Exposures”

**DOI:** 10.1289/EHP730

**Published:** 2016-11-01

**Authors:** Daniel Murphy, James Pay, Rebecca Benham, Benjamin James, David Hutchinson

**Affiliations:** 1 Rheumatology Department, Royal Cornwall Hospital, Truro, Cornwall, United Kingdom; 2 University of Exeter Medical School, Truro, Cornwall, United Kingdom

We read the paper by Parks et al. with interest, particularly the increased risk of rheumatoid arthritis (RA) reported for maneb and mancozeb pesticide use (odds ratio [OR] = 3.3; 95% confidence interval [CI]: 1.5, 7.1). We suggest that these findings are explained by heavy metal exposure.

Maneb and mancozeb fungicides are approximately 21% manganese by weight (Gunier et al. 2014). Their use is associated with increasing concentrations of manganese in house dust from Californian farm worker residences, and manganese concentrations decline with distance from use (Gunier et al. 2014). Mancozeb use results in raised local sediment concentrations of manganese, as demonstrated by long-term use in banana plantations (Melgar et al. 2008).

Other farm exposures may predispose individuals to metals inhalation. The authors report an association between RA and chemical fertilizer application (OR = 1.7; 95% CI: 1.1, 2.7). Phosphate fertilizers can contain cadmium as a contaminant in phosphate rock, with rates of cadmium contamination in soil ranging from 0.3 to 1.2 g/ha (Mortvedt 1987).

Of note, this group has previously demonstrated welding to be associated with RA in women (OR = 2.1; 95% CI: 0.8, 5.4) (De Roos et al. 2005), as welding fumes are associated with inhalation of both cadmium and manganese particles.

Furthermore, in the current study Parks et al. demonstrated increased RA risk with > 18 pack years smoking (OR = 1.5; 95% CI: 1.0, 2.2). Cadmium is among the hazardous components of cigarette smoke, and the mean whole blood cadmium concentration in smokers has been reported as double that of nonsmokers (2.67 ± 1.21 μg/L versus 1.37 ± 0.45 μg/L) (Bernhard et al. 2005).

We propose that inhalation of soil dust contaminated with manganese and cadmium may be a cause of RA. Cadmium exposure has been hypothesised as a trigger for RA, providing a common thread between smoking and many other known RA risk factors (Hutchinson 2015). This hypothesis is supported by a rat model that exhibited enhanced collagen-induced arthritis disease activity when exposed to high levels of cadmium (Ansari et al. 2015).

Inhalation exposure is relevant as the lung is an important initiating site of seropositive RA, due to local generation of anti-citrullinated peptide antibody (Perry et al. 2014). Cadmium and manganese have the potential to cause citrullination within the lung as calcium channel activators, significantly raising intracellular calcium levels (Hinkle et al. 1992).

Future studies investigating the link between RA, pesticides, and other farm exposures should consider exposures to metals as potential risk factors.
